# Normal Thymic Size and Low Rate of Infections in Human Donor Milk Fed HIV-Exposed Uninfected Infants from Birth to 18 Months of Age

**DOI:** 10.1155/2013/373790

**Published:** 2013-04-30

**Authors:** Dorthe Lisbeth Jeppesen, Annette Kjær Ersbøll, Tine Ursula Hoppe, Susanne Dam Nielsen, Niels Henrik Valerius

**Affiliations:** ^1^Department of Paediatrics, Unit 460, Hvidovre Hospital, University of Copenhagen, Kettegaard Alle 30, 2650 Hvidovre, Denmark; ^2^National Institute of Public Health, University of Southern Denmark, Øster Farimagsgade 5 A, 1353 Copenhagen K, Denmark; ^3^Department of Infectious Diseases, Unit 144, Hvidovre Hospital, University of Copenhagen, Kettegaard Alle 30, 2650 Hvidovre, Denmark; ^4^Department of Infectious Diseases, Rigshospitalet, University of Copenhagen, Blegdamsvej 9, 2100 Copenhagen Ø, Denmark

## Abstract

*Objective*. To evaluate the immune function in HIV-exposed uninfected (HIV-EU) infants fed human donor milk. *Methods*. Ultrasound-obtained thymic index (Ti), T-lymphocyte subsets, and the number of infections were examined from birth to 18 months of age in 18 HIV-EU infants. The infants were compared to a cohort of 47 term, HIV-unexposed breastfed or formula-fed infants. *Results*. The thymic size at 12 months of age was not significantly different between the HIV-EU group and the control infants (*P* = 0.56). At 4 months of age, the HIV-EU infants had significantly fewer infections than the control infants (*P* < 0.001). Furthermore, in the control group, the infants exclusively breastfed at 4 months of age had significantly fewer infections at 8 months when compared to age-matched formula-fed infants (*P* = 0.001). *Conclusion*. HIV-EU infants fed human donor milk have normal growth of thymus and contract fewer infections than other healthy infants. This finding along with fewer infections in exclusively breastfed infants compared to formula-fed infants supports the beneficial effect of human milk on the immune system. We suggest, when breastfeeding is not possible, that providing human donor milk to vulnerable groups of infants will be beneficial for their maturing immune system.

## 1. Introduction

Vertical transmission of HIV from HIV-positive mothers to their infants is, in industrialised countries, reduced to less than 1-2% [1–3]. Consequently, the number of HIV-exposed uninfected (HIV-EU) infants in the world is growing. Despite HIV-EU infants remaining uninfected, there have been reports of impaired immune function and reduced CD4 counts in HIV-EU newborns [4–8]. However, reports on the long-term impact of HIV exposure on the immune system have been conflicting and there have been very few studies of whether the infection burden in HIV-EU infants is higher than in HIV-unexposed infants [9, 10].

The thymus plays a key role in the development of a functional immune system, providing the environment for T-lymphocyte maturation and being a central organ for the development and maintenance of cell-mediated immunity. The thymus is also known to be a target organ in HIV-infection [11]. The transition of T-cell progenitors in the thymus has been extensively evaluated, but the significance of the size or alterations in the size of the thymus in infancy is still unclear [12–15].

The positive correlation between thymic size and weight and length at birth and during the first months of life is well known and infections are reported to result in decreasing thymic size [16–20]. We have also demonstrated that breastfed infants born at term have a larger thymus than term, formula-fed infants. Furthermore, feeding HIV-exposed uninfected (HIV-EU) infants with donor breast milk up to 4 months of age leads to a thymic size less than in breastfed infants, but larger than in exclusively formula-fed infants at the same age [21–23].

In HIV-infected children, thymic size is smaller compared to HIV-negative children, as reported in two African studies [24, 25]. Furthermore, in a study from Guinea-Bissau, a small thymic size at 6 months of age was a strong risk factor for mortality [26]. Therefore, the thymic size in early infancy might correlate to later immune function.

Few studies have managed to demonstrate an association between thymic size and T-lymphocytes. In the two African studies of HIV-infected children, the thymic volume was found to correlate positively with the CD4 count and negatively with HIV viral load [24, 25]. In a previous study of healthy mature infants followed up to 10 months of age, we demonstrated a correlation between thymic size and CD8+ cells [27]. In a clinical review of the best available evidence, 9 case-control studies of neonatal thymectomy in cardiac surgery were reviewed; neonatal thymectomy had a documented effect on T-cell numbers and distribution, and the CD4 count decreased after thymectomy in all the studies reviewed. However, lower CD4 counts in thymectomized children did not seem to lead to more infections in childhood [28].

The present study was conducted to evaluate the thymic size in HIV-EU infants compared to term HIV-unexposed infants. Furthermore, the impact of prenatal HIV exposure on HIV-negative infants fed with human donor milk in their first four months of life on the thymic size and frequency of infections during the first year of life was evaluated. Finally, we aimed to examine a possible correlation between ultrasound-obtained thymic size, T-lymphocyte subsets in peripheral blood, and the frequency of infections. 

## 2. Methods

### 2.1. Cohort

From December 1996 to October 1999, all infants born to HIV-positive mothers at Hvidovre University Hospital were invited to participate in the study. One mother declined to take part in the study. A cohort of 18 infants born to HIV-positive mothers was included in the study. The study population included one pair of twins. All but one infant were delivered by Caesarean section. The 17 HIV-positive mothers included 2 ethnic Danes (including 1 former intravenous drug user (IVDU)), 12 women from Africa, and 3 women from Asia. Antiretroviral treatment antepartum and/or intrapartum was accepted by all the HIV-positive mothers. None of the infants were infected with HIV as detected by PCR at the age of 6 months. One infant born to an HIV-positive mother was born preterm at a gestational age (GA) of 31 weeks (GA being the age of the infant determined as the number of weeks after the first day of the last menstrual period). The infants were examined at birth and after 2 (*n* = 18), 4 (*n* = 13), 8 (*n* = 11), 12 (*n* = 9), and 18 (*n* = 6) months of age. A capillary blood sample was taken at each examination. The clinical variables registered in all children were length, weightm and age. All information about infections was obtained from parental registered diaries during the observation periods. The parents were then interviewed by the same investigator and information concerning their child's previous illnesses was registered as number of episodes with otitis media, pneumonia, upper respiratory tract infection, wheezing, gastroenteritis, pharyngitis, and episodes of fever. Ongoing illness or illness during the preceding week of examination was registered. The cumulated sum of infectious events between the examination periods was analyzed. 

All HIV-EU infants were exclusively fed pasteurized human milk (donor milk) provided from the local milk bank: The Hvidovre Milk Bank, which is the largest milk bank in Denmark. The milk is obtained from mothers who are referred to the milk bank by health visitors. All donor milk mothers were initially interviewed at the milk bank and all were screened for HIV, hepatitis B, and hepatitis C. Milk from immigrants was screened for TB. The donor milk mothers come from all over the island of Sjaelland, include all ethnicities, and were paid per liter for their milk. All the milk was pasteurized and controlled according to protocols and guidelines that were part of the public health law. For further information please see the article by Arnold in 1999 [29].

The thymic size and clinical outcome in the HIV-EU infants were compared to a cohort of 47 term HIV-unexposed infants from an earlier thymus cohort study conducted in the period October 1994 to December 1996 [16, 17, 21]. These infants were all born to Danish mothers, except one whose mother was African. There were no IVDU among the mothers in the control cohort, but they came from the same Copenhagen suburbs as the HIV-EU infant's mothers. However, detailed data on socioeconomic status are not available. The HIV-unexposed infants were also examined at birth (*n* = 47) and reexamined at 4 (*n* = 47), 8 (*n* = 37), and 12 (*n* = 37) months of age. The HIV-unexposed infants were divided into three groups according to breastfeeding status at 4 months of age: exclusively breastfed, partially breastfed (breastfeeding plus formula feeding), and exclusively formula-fed. Information on weight, length, and infectious illness was collected at each examination in all the infants in the HIV-unexposed cohort in the same way as done in the HIV-EU infants [16, 17, 21]. There was no time-dependent difference in vaccination strategy and governmental day-care provision between the cohort of HIV-EU infants and the control cohort of HIV-unexposed infants.

### 2.2. Flow Cytometry

Blood samples were collected and used for analysis of lymphocyte subsets by flow cytometry. A total leukocyte count and a differential white cell count were obtained to calculate the absolute numbers of lymphocyte subsets. Flow cytometry was performed as described previously [5]. Briefly, 50 *μ*L blood was incubated with 10 *μ*L fluorescence-conjugated monoclonal antibodies at room temperature for 15 minutes. Erythrocytes were lysed with 2 mL NH_4_Cl buffer at room temperature for 10 minutes, then the samples were washed and resuspended in phosphate-buffered saline supplemented with 10% CellFix (Becton Dickinson Immunocytometry Systems (BD), San Jose, CA, USA). All samples were analyzed using a FACScan (BD) equipped with a 488-nm argon-ion laser. Data were processed using CellQuest software (BD). Monoclonal antibodies used to determine phenotypes were isotype controls, CD3 (Leu-4), CD4 (Leu-3a), and CD8 (Leu-2a). Immature thymocyte precursors were determined using CD7 (Leu-9) and CD1a (Leu-6). All antibodies were purchased from BD. 

Lymphocyte subsets were not analysed in the control cohort.

### 2.3. Thymic Size

The size of the thymus was assessed by ultrasound as the thymic index (Ti) as previously described by Hasselbalch et al. [17]. By ultrasound, the thymus appears as a well-delineated echo-poor structure in the anterior mediastinum. Using a transsternal approach, the largest transverse diameter (cm) was first obtained. Perpendicular to that, the largest sagittal area was found and measured (in cm^2^). Each measurement was obtained twice, and if difference of more than 15% was found, both measurements would be repeated. Ti was calculated by multiplying the transverse diameter by the sagittal area. 

### 2.4. Statistics

Descriptive analysis was performed by means of mean and median values and range (i.e., mix–max) for Ti, weight, length, and cumulated number of infections and T-lymphocytes. 

The correlation between Ti, weight, length, and cumulated number of infections and T-lymphocytes was calculated using Spearman's correlation coefficient. 

Differences in Ti at birth and at 12 months of age between the HIV-EU and the HIV-unexposed infants were evaluated using an analysis of variance. Ti was the outcome variable and exposure (yes, no) was included as a fixed effect. Due to a skewed distribution of Ti, a logarithmic transformation of Ti values was used in the statistical analysis. The assumptions were evaluated using the Shapiro-Wilks test for a normal distribution and visual inspection of residual plots.

A repeated measurements analysis of variance was performed to evaluate differences in Ti between feeding from 0 to 12 months of age. Ti was the outcome variable. Feeding group and month were included as fixed effects. Differences in the development of Ti between feeding groups during the study period were evaluated by including the interaction between month and feeding group. The autocorrelation between repeated Ti values for the same infant was taken into account by including an autoregressive correlation structure in the model. The assumptions were evaluated using the Shapiro-Wilks test for a normal distribution and visual inspection of residual plots. 

The association between the cumulated number of infections and Ti was evaluated in a repeated measurements analysis with cumulated number of infections as the outcome variable. A Poisson regression model was used. Ti, month, and feeding group were included as fixed effects. The autocorrelation between repeated numbers of infections for the same infant was taken into account by including an autoregressive correlation structure in the model. The dispersion parameter was used to evaluate the goodness-of-fit for the model. 

The association between T-lymphocyte subsets and Ti was analyzed using a repeated measurements analysis of variance with each of the T-lymphocyte subgroups as the outcome variable. Ti and month were included as fixed effects. The autocorrelation between repeated T-lymphocyte values for the same infant was taken into account by including an autoregressive correlation structure in the model. The assumptions were evaluated using the Shapiro-Wilks test for a normal distribution and visual inspection of residual plots. 

A 5% significance level was used in all analyses. All statistical analyses were performed using SAS Statistical Analysis Software (version 9.2). 

## 3. Results

### 3.1. The Thymic Size in HIV-EU Infants Compared to Term HIV-Unexposed Infants When Not Taking Feeding Mode into Account

Ti in HIV-EU infants and in the cohort of term HIV-unexposed infants is shown in Tables 1 and 2, respectively. There was a significant positive correlation between Ti and weight and between Ti and length in HIV-EU infants from birth up to 18 months (for both: *r* = 0.64, *P* < 0.001). 

**Figure 1:**
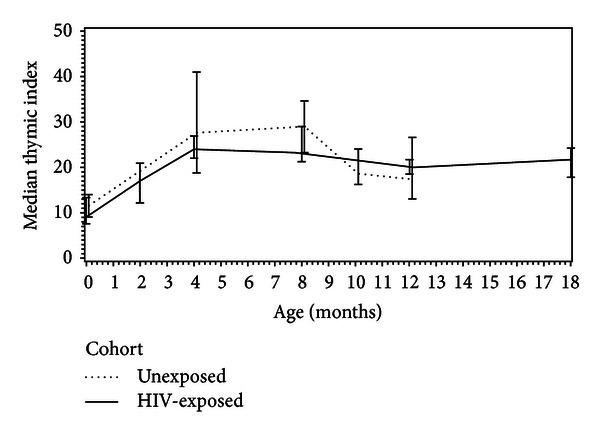
The thymic size in HIV-EU infants compared to term HIV-unexposed infants. In general, there is no difference in thymic size between the cohorts from birth to 12 months of age when feeding mode is not included in the analysis. The Ti is given as median and with Q_1_  and Q_3_.

The thymic size in the HIV-EU infants at birth tended to be lower than thymic size in the term infants at birth, although this did not reach significance (*P* = 0.08). The thymic size at 12 months of age was not significantly different between the HIV-EU and the total control cohort (*P* = 0.56) (Figure 1). 

### 3.2. Impact of Different Feeding Mode on Thymic Size

**Figure 2:**
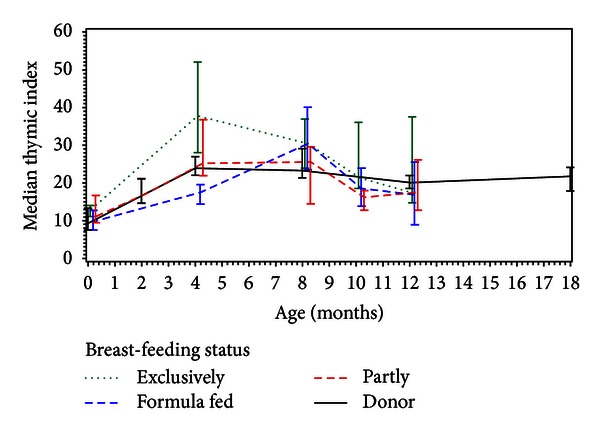
Feeding mode at 4 months of age had a significant impact on the thymic size. Those breastfed at 4 months of age have the largest thymus when compared to the three other feeding groups. However, the HIV-EU infants at 4 months of age have a thymic size the same as the partially breastfed infants. By 8- and 12-month followups the difference in Ti between the feeding groups disappeared. Ti is given as medium and with Q_1_  and Q_3_.

There was a significant difference in Ti between the four feeding groups across the examination period 0–12 months of age (*P* = 0.005 for the interaction between age and feeding mode). The thymic size at 4 months of age was significantly smaller in the HIV-EU infants than in the exclusively breastfed infants (*P* = 0.002) (Figure 2). However, the thymic size at 4 months of age in the HIV-EU infants tended to be greater than in those exclusively formula-fed (*P* = 0.06), but not significantly different from Ti in the partially breastfed infants (*P* = 0.45). There was no significant difference in Ti between the feeding groups at the 8- and 12-month followups. Ti in the different feeding groups in the cohort of term HIV-unexposed infants is shown in Table 3.

### 3.3. The Thymic Size and Frequency of Infections in HIV-EU Infants

There was no overall correlation between Ti and number of infections at 0, 4, 8, 12, and 18 months of age (*r* = 0.14, *P* = 0.32).

### 3.4. The Frequency of Infections in HIV-EU Infants Compared to the Different Feeding Groups of the Term HIV-Unexposed Infants

There was a significant difference in the cumulated number of infectious events between the feeding groups across the examination period 0–12 months of age (*P* < 0.001 for the interaction between age and feeding mode). Between birth and 4 months of age, there was no significant difference in the cumulated number of infectious events between the HIV-EU infants and each of the HIV-unexposed feeding groups (exclusively breastfed *P* = 0.81, partially breastfed *P* = 0.98, and formula-fed *P* = 0.44, resp.). Between 4 and 8 months of age, the HIV-EU infants had significantly fewer infections than the HIV-unexposed exclusively breastfed (*P* < 0.001), partially breast fed (*P* < 0.001), and formula-fed (*P* < 0.001) infants. At the 12-month examination, the HIV-EU infants still had significantly fewer infections than each of the feeding groups in the HIV-unexposed infants at 12 months of age (exclusively breastfed *P* < 0.001, partially breastfed *P* = 0.008, and exclusively formula-fed *P* = 0.001, resp., Table 4). 

Among the HIV-unexposed infants, the exclusively breastfed infants had significantly fewer infectious events at 8 months of age than the formula-fed infants at the same age (*P* = 0.001), but were not significantly different from the partially breast fed HIV-unexposed infants (*P* = 0.15). The mean number of cumulated infectious events per child is shown in Table 4. 

### 3.5. The Association between Thymic Size and T-Lymphocyte Subgroups in HIV-EU Infants

Table 5 shows the result of T-lymphocyte counts. The numbers of CD1a+ CD7+ immature thymocyte precursors were positively associated with the thymic size (*P* = 0.01), an increase in the numbers of CD1a+ CD7+ immature thymocyte precursors leading to a greater thymic size. The association was most significant in the younger age group with a *P* value at the 4-month examination at 0.001. No other association between thymic size and T-lymphocyte subsets was observed.

### 3.6. Frequency of Infections in HIV-EU Infants Compared to the T-Lymphocyte Subgroups

A high number of CD3+, CD8+, and CD4+ cells was significantly associated with fewer infectious events during the observation period (*P* = 0.0007, *P* = 0.01, and *P* = 0.005, resp.).

## 4. Discussion

The purpose of this study was to evaluate the thymic size and infections in HIV-EU human donor milk fed infants compared to term HIV-unexposed infants. In general, equal growth and size of thymus was found in the HIV-EU infants compared to term HIV-unexposed infants. Interestingly, HIV-EU infants had fewer infections up to one year of life, when compared to the HIV-unexposed infants. Finally, in the HIV-unexposed infants we demonstrated fewer infections when exclusively breastfed at 4 months of age. 

This birth cohort of HIV-EU infants was unique in Danish settings, being followed prospectively and given human donor bank milk for their first 4 living months. The possibility of giving donor milk to HIV-EU infants had ceased in 2003, because of high economic cost.

The thymic size was evaluated by ultrasound. The method was first described by Hasselbalch et al., and although thymic index is a volume estimate and not a true volume, it was shown by a postmortem study to correlate well with actual thymic volume [17]. The method is internationally accepted and has been used in several studies as a possible surrogate marker for immune function [10, 16, 20–23, 26, 27].

At birth, the thymic size tended to be smaller in the cohort of HIV-EU infants (when the preterm infant was included) compared to the cohort of HIV-unexposed infants. However, when feeding mode was not taken into account the thymic size at one year of age in this cohort of HIV-EU infants did not differ significantly from the whole cohort of term healthy HIV-unexposed infants at the same age.

This is in contrast to a recent study of HIV-EU infants examined at around 15 months of age, who had a significantly smaller thymic size compared to the matched cohort of infants born to HIV-negative mothers [10]. The equal size and growth of thymus in the HIV-EU infants could be due to ethnicity the thymic size at birth being relatively larger in the HIV-EU, as 12 of 18 infants were born to African mothers. Thus, an African study showed a “marginally” larger thymus at birth in African children compared to Danish children [30].

These conflicting findings might be explained by the different feeding modes of the two cohorts of HIV-EU infants; the infants in this presented study received human donor milk in the first 4 months of life, compared to the other cohort of HIV-EU infants who were exclusively formula-fed from birth [10]. This finding is also supported by the fact that the growth curve for thymus in the HIV-EU children in the presented study was similar to the growth curve of thymus in partially breastfed infants as seen in Figure 2. A similar growth pattern of thymus was seen in a recent study of preterm infants (offered human donor milk from birth and having a high breastfeeding rate later on), in which the thymus was very small at birth, but comparable in size at one year of age compared to term infants at one year of age [20]. The excellent growth of thymus may in both cases be caused by the high intake of breast milk. 

Interestingly, HIV-EU infants had significantly fewer infections up to one year of life when compared to term HIV-unexposed infants. This finding may be explained by the feeding mode too, but even compared to the exclusively breastfed infants the rate of infections was lower. Another potential explanation could be differences in attending day-care or number of older siblings between the two cohorts. Unfortunately, we do not have data on day-care attendance or number of siblings. Also, the lower the number of infections in the HIV-EU infants could be explained by different socio-economic status, but the mothers from both cohorts came from the same suburbs of Copenhagen. Finally, the ethnical difference between the HIV-EU and the control infants might influence both occurrences of infections and thymic growth, but to our knowledge no Danish studies have shown difference in occurrence of infections between different ethnic groups of infants in Denmark. 

It could be discussed whether parental recall for infections is reliable [31], but a recent Danish study by Vissing et al. supports the validity of Danish parental interviews in longitudinal cohort studies investigating atopic disease and illness in childhood [32]. Also, the time interval over which the parents had to recall information was, at most, the previous 4 months.

One might speculate that being exposed to HIV-virus in utero might prime the immune system in the same way as vaccines are suggested to have immune modulation properties influencing child mortality in African infants [26, 33], but this has to be further investigated in a future study. For the first time in Denmark we have demonstrated that being exclusively breastfed at 4 months of age compared to exclusively formula-fed at the same age leads to significantly fewer infections up to 8 months of age. This supports the possible immune modulating and positive effect of being breastfed up to a minimum of 4 months of age, for infants in westernised as well as developing countries. Therefore, the decision to advise against breastfeeding should be carefully considered, especially in developing countries, where the infectious burden is much higher [33].

The correlation between CD4+ and CD8+ cells and thymic size demonstrated in previous studies was not observed in this study [27]. Instead, a strong association between the CD1a+ CD7+ immature thymocytes and the thymic size was observed up to 12 months of age. We suggest that this might be due to a higher thymic output in infants with a larger thymus. Finally, an inverse correlation between number of infections and the total number of both CD8+ and CD4+ T-lymphocytes was observed. One interpretation of this finding could be that a high number of T-lymphocytes in the HIV-EU infants leads to a reduced sensitivity to infection. However, since T-lymphocytes in the controls were not analysed this finding should be interpreted with caution, and further studies are warranted.

## 5. Conclusion 

This study shows that HIV-EU infants fed human donor milk are capable of a normal growth of thymus and contract fewer infections than other infants. Furthermore, the finding of fewer infections at 8 months of age in the HIV-unexposed infants who were exclusively breastfed to 4 months supports evidence for a beneficial effect of human milk on the immune system. We suggest that when breastfeeding is not possible, that providing vulnerable groups of infants human donor milk will be beneficial for the maturing immune system.

## Figures and Tables

**Table 1:** HIV-exposed un-infected infants (HIV-EU): the number of infants, the thymic size (thymic index (Ti)), and the weight and length at the times of assessment.

Time of examination	Newborn	2 months	4 months	8 months	12 months	18 months
Number	18	13	13	10	9	6
Ti (median)	9.3 (5.4–21.0)	17.2 (11.9–33.9)	24.0 (15.9–31.1)	24.3 (18.0–36.7)	20.0 (15.3–28.2)	21.8 (8.5–26.0)
Weight, (g) mean (range)	2926 (1790–4000)	4159 (3390–5950)	6005 (4700–7900)	8216 (7050–11050)	9311 (8250–10350)	10660 (9700–12700)
Length, (cm) mean (range)	49.7 (43–54)	54.7 (50.5–59.0)	60.7 (54.0–66.0)	68.2 (62.5–73.5)	74.2 (68.5–78.5)	77.9 (75.0–81.5)

**Table 2:** The term HIV-unexposed infants: the number of infants, the thymic size (Ti), and the weight and length at the times of assessment.

Time of examination	Newborn	2 months	4 months	8 months	12 months
Number	47	Not examined	47	37	37
Ti (median)	11.8 (3.8–23.9)	Not examined	27.7 (12.2–83.2)	29.0 (6.3–55.2)	17.3 (7.4–53.0)
Weight, (g) mean (range)	3524 (2550–4850)	Not examined	7091 (5090–9476)	9222 (7100–11389)	10442 (8259–12359)
Length, (cm) mean (range)	52.0 (49.0–57.0)	Not examined	64.7 (59.0–69.0)	72.3 (66.0–78.0)	76.9 (68.0–82.0)

**Table 3:** The term HIV-unexposed infants divided into the three feeding groups at 4 months of age: the number of infants, the thymic size (Ti, median, and range), and the weight and length (mean and range) at the times of assessment.

Three feeding groups	Values	Newborn	4 months	8 months	12 months
Exclusively breastfed	Number	21	21	15	15
Exclusively breastfed	Ti	12.9 (7.0–23.9)	37.8 (16.2–83.2)	30.6 (16.8–55.2)	17.3 (9.9–53.0)
Exclusively breastfed	Weight (g)	3446 (2750–4200)	7237 (5090–8967)	9262 (7147–11384)	10375 (8522–11947)
Exclusively breastfed	Length (cm)	52.1 (50.0–55.0)	64.9 (59–69)	72.3 (66.0–76.50)	77.7 (68.0–82.0)
Partially breastfed	Number	13	13	11	11
Partially breastfed	Ti	11.2 (5.5–21.1)	25.2 (15.6–50.0)	25.6 (6.3–53.1)	17.4 (9.3–31.1)
Partially breastfed	Weight (g)	3658 (2600–4500)	6917 (5333–8850)	8887 (7100–11200)	10114 (8601–12149)
Partially breastfed	Length (cm)	52.7 (49.0–57.0)	64.3 (60.0–68.2)	71.3 (67.0–76.0)	75.4 (71.5–80.0)
Exclusively formula-fed	Number	13	13	11	11
Exclusively formula-fed	Ti	9.7 (3.8–20.2)	17.6 (12.2–32.6)	30.4 (7.4–47.6)	17.0 (7.4–33.0)
Exclusively formula-fed	Weight (g)	3518 (2550–4850)	7029 (5225–9476)	9503 (7282–11389)	10861 (8259–12359)
Exclusively formula-fed	Length (cm)	52.4 (49.0–56.0)	64.7 (60.0–69.0)	73.2 (70.0–78.0)	77.5 (74.0–81.0)

**Table 4:** Events of infectious illness in HIV-exposed uninfected (HIV-EU) infants compared to term HIV-unexposed infants, according to their different feeding modes at 4 months of age. The observation period between each examination was 4 months. *N* is the mean number of cumulated infectious events between two evaluations per child in every feeding mode category.

	Mean number (min–max) of cumulated infectious events		
Time of examination:	4 months	8 months	12 months
Feeding mode and cohort:			
HIV-EU: human donor milk	1.8 (0–8)	1.7 (0–4)*	3.2 (0–7)*
HIV-unexposed infants: exclusively breastfed until 4 months of age	2.0 (0–4)	5.7 (1–13)**	9.0 (5–20)
HIV-unexposed infants: exclusively formula-fed until 4 months of age	2.5 (1–5)	12.5 (2–35)	9.5 (3–15)
HIV-unexposed infants: partially breastfed until 4 months of age	1.8 (1–3)	8.2 (1–26)	7.9 (4–13)

*HIV-EU infants had significantly less infectious events than the other infants.

**Infants exclusively breastfed until 4 months of age had significantly fewer infections at 8 months of age, than the exclusively formula-fed infants.

**Table 5:** Distribution of T-lymphocyte subsets in HIV exposed uninfected infants given as median and range (min–max) and the number of infants (*n*). Cell count is given in cell/*μ*L.

T-lymphocytes	Newborn (*n* = 16)	2 months (*n* = 12)	4 months (*n* = 11)	8 months (*n* = 11)	12 months (*n* = 8)	18 months (*n* = 6)
CD3+	2575 (839–4300)	3340 (2094–5281)	3340 (1995–4697)	3451 (2548–5059)	3433 (2043–5396)	3825 (1686–5073)
CD4+	1109 (325–2157)	1370 (803–2855)	1468 (83–2049)	1426 (962–1967)	1325 (849–2502)	1263 (658–2488)
CD8+	463 (65–2895)	642 (306–2991)	467 (128–1264)	634 (263–1708)	631 (242–1423)	551 (254–902)
CD1a+ CD7+	9.1 (3.6–65.8)	26.2 (3.8–126.2)	26.5 (10.4–114.6)	25.3 (6.1–83.5)	30.1 (7.0–98.9)	17.7 (8.3–69.0)
